# Integrated transcriptome and metabolome analysis reveals a possible mechanism for the regulation of lipid metabolism via vitamin A in rice field eel (*Monopterus albus*)

**DOI:** 10.3389/fphys.2023.1254992

**Published:** 2023-08-23

**Authors:** Huanhuan Huo, Chonghua Hu, Qiubai Zhou, Liufeng Xiong, Mo Peng

**Affiliations:** ^1^ College of Animal Science and Technology of Jiangxi Agricultural University, Nanchang, China; ^2^ Key Laboratory of Featured Hydrobios Nutrition Physiology and Healthy Breeding, Nanchang, China; ^3^ Ganzhou Animal Husbandry and Fisheries Research Institute, Ganzhou, China

**Keywords:** *Monopterus albus*, vitamin A, transcriptome, metabolome, lipid metabolism

## Abstract

To understand the effects of vitamin A on lipid deposition in rice field eels, integrated liver transcriptome and metabolome were conducted and the changes in the genes and metabolites were assessed. Three groups of rice field eel were fed with 0, 200, and 16,000 IU/kg vitamin A supplementations in their diets for 70 days. The total lipid content in the whole body of the rice field eels was significantly increased with the vitamin A supplementations (*p* < 0.05). Comparative transcriptome analysis revealed 14 pathways and 46 differentially expressed genes involved in lipid metabolism. Sphingolipid metabolism, glycerolipid metabolism, primary bile acid biosynthesis and steroid hormone biosynthesis were significantly enriched pathways. In these pathways, three differential genes phospholipid phosphatase 1a (*PLPP1a*), phospholipid phosphatase 2b (PLPP2b), cytochrome P450 21a2 (*CYP21a2*) were consistent with the change trend of lipid content, and the other three differential genes aldo-keto reductase family 1 member D1 (*AKR1D1*), uridine diphosphate glucuronic acid transferase 1a1 (*UGT1a1*), cytochrome P450 1a (*CYP1a*) were opposite. Metabolomic analysis revealed that primary bile acid biosynthesis, sphingolipid metabolism, steroid hormone biosynthesis and biosynthesis of unsaturated fatty acids were all critical for rice field eel metabolic changes in response to vitamin A. Six important differential metabolites (eicosapentaenoic acid, sphinganine, 11-beta-hydroxyprogesterone, hydroxyeicosatetraenoic acid, cholic acid, and glycochenodeoxycholate) were identified and have provided new insights into how vitamin A regulates lipid deposition. Integrated transcriptome and metabolome analyses revealed that primary bile acid biosynthesis was the only remarkably enriched pathway in both the transcriptome and metabolome while that sphingosine was the main metabolite. Based on the above results, we have concluded that vitamin A promotes lipid deposition in the rice field eel through the primary bile acid synthesis pathway, and lipid deposits are widely stored in cell membranes, mainly in the form of sphingosine. These results will provide reference data to help improve our understanding of how vitamin A regulates lipid metabolism.

## 1 Introduction

Vitamin A (VA) is an essential nutrient for fish that helps to maintain growth and development as it is involved in various physiological reactions in the body including lipid metabolism (Roels, 1969). Studies have shown that the effects of VA on lipid metabolism have species-dependent effects. For example, the crude lipid contents in herring (Lian et al., 2017), largemouth bass (Chen et al., 2020) and sea bass (Zhang et al., 2015) were found to increase with increasing dietary VA levels, and then become stable. In contrast, the whole-body crude lipid content levels of Atlantic salmon (Ørnsrud et al., 2002), groupers (Mohamed et al., 2003) and juvenile flounder (Hernandez et al., 2005) were found to significantly decrease with increases in dietary VA. Furthermore (Thompson et al., 1995) found that VA intake had no significant effect on the body composition of rainbow trout and (Yu et al., 2019) found that VA had no significant effect on the crude lipid content of whole tilapia fish.

There are many mechanisms by which VA regulates lipid metabolism, including gene transcription factors that regulate lipid synthesis and lipid oxidation, signaling pathways related to lipid metabolism, number of adipocytes, secretion of adipocytokines, and epigenetic modifications in mammals (Wang and Yan, 2017). Peroxisome proliferator activated receptors (PPARs) are a class of lipid-activated transcription factors that regulate lipid metabolism. VA deficiency can decrease transcription levels of PPARβ, resulting in increased levels of polyunsaturated fatty acids, such as linoleic, linolenic, arachidonic, and docosahexaenoic (Yang et al., 2005). The activation of PPARα can be inhibited by the liver X receptor -sterol regulatory element-binding protein-1c pathway which downregulates the expression of fat synthesis genes, and the activation of liver X receptor inhibits PPARα-induced fatty acid oxidation (Tomohiro et al., 2003). Studies have suggested that VA promotes fat synthesis, which may be associated with epigenetic modifications. When VA is present, polycomb repression complexes can rapidly induce the separation of the zinc finger protein 423 (*Zfp423*) promoter, demethylation of the *Zfp423* histone, and the expression of *Zfp423* to promote fat synthesis (Wang et al., 2016). Kim et al. (2013) showed that the Wnt/β-catenin signaling pathway is involved in the differentiation of 3T3-L1 preadipocytes and that all-trans-retinoicacid inhibits the differentiation of 3T3-L1 preadipocytes through the transcriptional activation of β-catenin, which affects fat synthesis. The effects of VA on animal lipid metabolism are not consistent, as the mechanism is complex and there are organizational differences, and further research is thus required.

The *Monopterus albus* belongs to the genus eels of synbranchiidae and it is an economically important species in China. In 2022 years, its annual production was 334215 tons. The regulation of lipid levels by VA in rice field eels, however, has not yet been reported. Therefore, this study aims to explore the possible mechanism of VA-mediated regulation of lipid metabolism in rice field eels through comprehensive analysis of transcriptomics and metabolomics.

## 2 Materials and methods

### 2.1 Diet preparation

The fundamental feed formula is shown in Table 1. Fish meal was used as main protein sources. VA acetate (500,000 IU/g, Maclin Biochemical Co., Ltd., Shanghai, China) is the supplemental source of VA. Three isonitrogenous and isolipidic diets were formulated adding VA 0, 200 and 16000 IU/kg (3 201, 4 730 and 16890 IU/kg, actually) denoted as LA, MA and HA groups respectively. All raw materials were screened for 80 mesh and thoroughly mixed. After adding 16% moisture, the pellets were made by the pelletizer at 120°C. After air drying, the feed were stored at −20°C.

**TABLE 1:** Composition and nutrient levels of the basal diet(DM basis).

Ingredients	Content (%)
Fish meal	45
Chicken powder	8
Corn protein powder	4
Soybean meal	10
Wheat meal	22.5
Soybean oil	2
Compound protein1)	3
Lecithin	2
Ca(H2PO4)2	2
Premix2	1.5
Nutrient levels3	
CP	48.36
EE	7.80
Moisture	5.67
Ash	11.11

Note: the compound protein is made of soybean protein concentrate, earthworm powder and shrimp powder. 2) The premix provided the following per kg of the diet:VD3 2 500.0 IU, VE 200.0 mg, VK3 10.0 mg, VB1 25.0 mg, VB2 45.0 mg, nicotinic acid 200.0 mg, VB6 20.0 mg,Ca-pantothenate acid 60.0 mg, folic acid 10.0 mg, VB12 0.1 mg, biotin 1.5 mg, VC 200.0 mg, inositol 200.0 mg, NaSeO3•5H2O 0.3 mg, CoCl2•6H2O 0.4 mg, KI 0.8 mg, CuSO4•5H2O 10.0 mg, MnSO4•4H2O 20.0 mg, ZnSO4•H2O 50.0 mg, FeSO4•7H2O 150.0 mg, MgSO4•7H2O 500.0 mg, NaCl 1 000.0 mg. 3) measured value.

### 2.2 Chemicals and reagents

Concentrated sulfuric acid, boric acid, hydrochloric acid, sodium hydroxide, petroleum ether, methanol, acetonitrile acetone, sodium sulfate and anhydrous ethanol purchased from Nanchang Jinsha chemical raw materials Co. LTD. Trizol reagent, electrophoretic buffer, Reverse transcription kit, loading buffer and SYBR Premix Ex Taq kite purchased from Jiangxi Guanyin Biotechnology Co. LTD.

### 2.3 Experimental animals and sample collection

The juvenile *Monopterus albus* were artificially bred by our team. All fish were reared temporarily for 14 days to acclimate to the conditions. After fasting for1 day, 480 fish (7.3 ± 0.02 g) were randomly assigned to 12 separate tanks of 3 groups (HA, MA, and LA, four repetitions per group) with 40 fish per tank. Each tank was filled with the right amount of fresh grass, *Eichhornia crassipes*. Fish were fed 3–5% of the body weight once daily at 18:00 p.m. A tuck net removed the feces and residual feeds. The breeding experiment lasted for 10 weeks and replaced with clean water weekly. The environmental conditions were: water depth 0.4 m; temperature, 28°C ± 1°C; dissolved oxygen >5.0 mg/L.

After the feeding trial, the fish were anaesthetized using MS222. Three fish from each tank were sampled randomly. The liver from twelve fish each group were dissected into 1.5 mL tubes and stored in −80°C for further RNA extraction. Three biological replicates were used for transcriptome sequencing and six biological replicates were used for metabolome detection. The remaining three fish each group were used to detect body composition.

### 2.4 Effects of dietary VA level on *Monopterus albus*

The crude protein, crude lipid, moisture and ash in whole fish body were measured following the methods of the Association of Official Analytical Chemists. The crude protein was determined by Kjeldahl method (FOSS, Kjeltec TM 8200). The crude lipid was estimated by using Soxhlet extraction method (FOSS, Soxtec 2050). The moisture was determined by drying samples in an oven at 105°C until constant weight. The ash was determined by combustion in a muffle furnace at 550°C for 4 h.

### 2.5 RNA extraction and transriptome sequencing

Liver RNA was extracted using Trizol reagent (Invitrogen, CA, United States) according to manufacturer’s instructions. Agarose gel electrophoresis was used to monitor RNA degradation. The Nano-Drop 2000 spectrophotometer was used to detect RNA concentration and purity. Further, the Agilent 2100 RNA 6000 Nano kitwas used to assess RNA integrity. Higher quality RNA was selected for high-throughput sequencing. Three RNA replicates of each sample were used to build RNA-seq library. After total RNA was extracted, the first strand cDNA was synthesized by random hexers and reverse transcriptase, and the second strand cDNA was synthesized by DNA polymerase I and RNase H. Then, a cDNA library was constructed for sequencing. The cDNA library was sequenced using Illumina HiSeqTM4000 by Gene Denovo Biotechnology Co. (Guangzhou, China). The transcriptome results were compared pair-to-pair.

### 2.6 Transriptome experimental validation using qPCR

Six genes (Table 2) were selected randomly for validation of RNA-Seq data by qPCR using a SYBR Premix Ex Taq kit (Invitrogen) according to the manufacturer’s instructions. The same RNA samples were used for both Illumina library synthesis and the qPCR verification assay. The first strand cDNA was obtained from 2 μg of total RNA using a PrimeScript first strand cDNA synthesis kit (Takara, Dalian, China). Melting curve analyses were performed following amplification. The specific primers used for qPCR are listed in Additional Table 2, and 18 s gene was used as an endogenous control. The thermal profile for SYBR Green qPCR was 95°C for 90 s, followed by 40 cycles of 95°C for 5 s, 60°C for 15 s, 72°C for 20 s.

**TABLE 2:** The information of primers used to qPCR.

Genes		5′-3′	Tm(°C)	Fragment size(bp)
*IL-1β*	F	GAG​ATG​TGG​AGC​CCA​AAC​TT	56.9	85
	R	CTG​CCT​CTG​ACC​TTC​TGG​ACT​T	58.5	
*Nkx6.1*	F	GGA​CAA​AGA​TGG​GAA​ACG​AAA	56.7	96
	R	GCC​AGG​TAT​TTG​GTC​TGT​TCA	58.2	
*RPL-17*	F	GTT​GTA​GCG​ACG​GAA​AGG​GAC	57.7	160
	R	GAC​TAA​ATC​ATG​CAA​GTC​GAG​GG	56.4	
*PCGF1*	F	CAG​CCC​TTA​CTC​AAC​CTC​AAA	57.9	167
	R	GCA​TCT​GGC​ACA​GCA​TCT​ACG	61.7	
*IGFBP-1*	F	CAG​AGA​GCC​TTG​GAA​AAG​ATT​G	57.3	171
	R	CTT​GCC​GTT​CCA​GGA​GTG​T	59.9	
*H3.3*	F	ATT​TTG​AGT​TGC​GGC​GAT​TA	56.4	181
	R	GTA​ACG​ATG​GGG​CTT​CTT​CAC	59	
*18S*	F	GTG​GAG​CGA​TTT​GTC​TGG​TTA	57.8	162
	R	CGG​ACA​TCT​AAG​GGC​ATC​AC	57.7	

### 2.7 Metabolites extraction LC-MS/MS analysis

The Liver samples were thawed at 4°C, appropriate samples were added into the pre-cooled methanol/acetonitrile/water solution (2: 2, v/v), vortex mixing, ultrasonic for 30 min, standing at −20°C for 10 min. After 14,000 g at 4°C for 20 min, supernatant vacuum drying, adding 100 μL acetonitrile solution during mass spectrometry (acetonitrile: Water = 1:1, v/v) redissolved, vortex, 14,000 g centrifuged at 4°C for 15 min. Then, supernatant was taken for analysis. Analysis was performed using an UHPLC (1290 Infinity LC, Agilent Technologies) coupled to a quadrupole time-of-flight (AB Sciex TripleTOF 6600) in Shanghai Applied Protein Technology Co.,Ltd. The metabolome results were compared in pairs.

### 2.8 The integrated analysis of transcriptomic and metabolomics data

Firstly, differential genes and differential metabolites related to lipid metabolism were identified by KEGG pathway analysis. Secondly, O2PLS (two-way orthogonal projections to late structures) model was established to analyze the relationship between them. Finally, pearson correlation coefficients were used to assess the correlation between differential genes and differential metabolites.

### 2.9 Statistical analysis

The results were expressed as mean ± SD. (standard deviation of the mean) and were analyzed using one-way ANOVA in SPSS 19.0 software. Any significant differences were further investigated by comparing the group means using Turkey’s test. Statistical significance was considered if *p* < 0.05.

## 3 Results

### 3.1 Effects of dietary VA levels on *Monopterus albus*

The crude lipid content in the whole body was significantly different (*p* < 0.05) among the different treatments, and that in the whole body of the LA group was significantly lower than that of the MA and HA groups (Table 3). The crude lipid content of the whole body increased with increasing dietary VA levels. In contrast, the differences in crude protein, moisture, and ash in the whole body were not significant.

**TABLE 3:** Effects of dietary VA levels on the nutritional composition of whole fish.

Whole body Index(%)	Groups		
	LA	MA	HA
Moisture	74.29 ± 0.35	73.53 ± 0.25	73.35 ± 0.14
Crude lipid	5.67 ± 0.12^a^	6.17 ± 0.19^b^	6.44 ± 0.07^b^
Crude Protein	16.81 ± 0.19	16.92 ± 0.01	17.12 ± 0.16
Ash	2.47 ± 0.01	2.47 ± 0.01	2.47 ± 0.02

Note: values represent means ± SD of four replicates. Values in the same row with different lowercase letters are significantly different (*p* < 0.05).

### 3.2 Transcriptomic analysis

**FIGURE 1:**
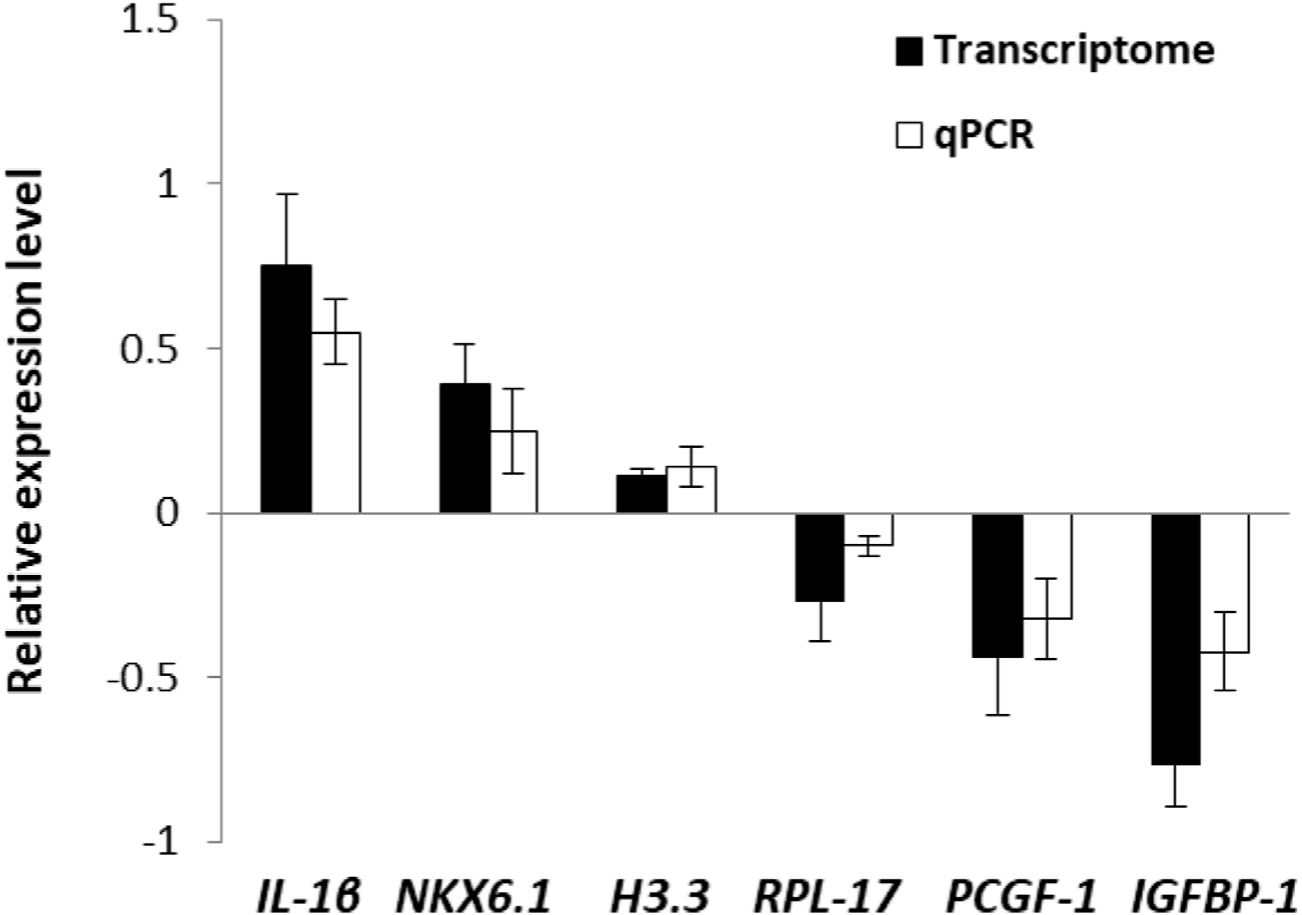
Validation of transcriptome data by using qPCR.

To validate the transcriptome data, 6 DEGs were selected randomly for qPCR. Althought the test DEGs displayed different expression levels (Figure 1), in general, the qPCR results showed a positive correlation with transcriptome, indicating the reliability and accuracy of the transcriptome analysis.

**FIGURE 2:**
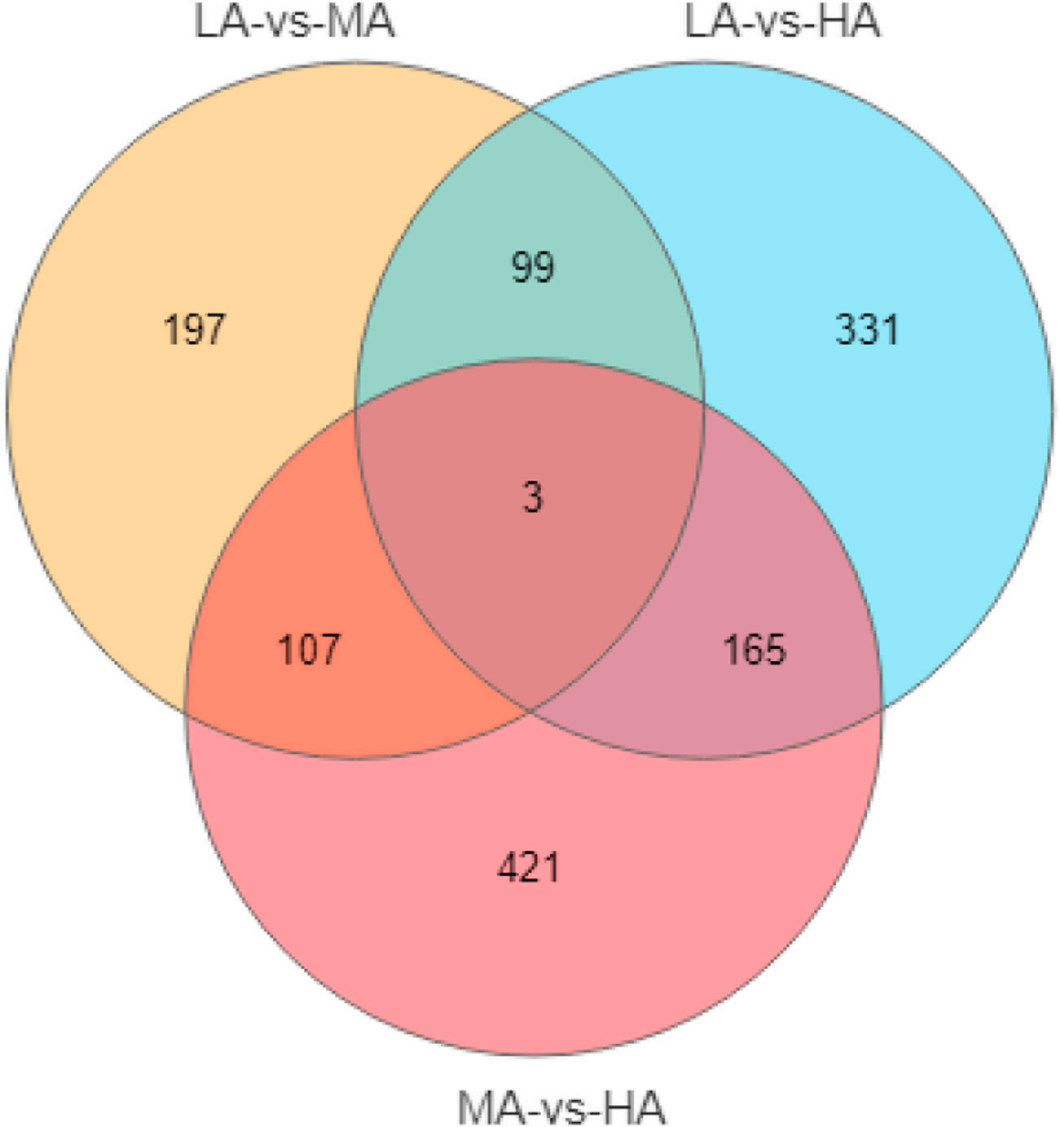
The venn of DEGs distribution in three groups.

After removing the low-quality reads, 50.52 million clean reads were obtained. A total of 32.0 Gb of data were generated by Illumina sequencing. The results of transcriptome have been submitted to NCBI (SRA accession no. SRP398570).Comparative transcriptome analysis identified 1323 differentially expressed genes (DEGs) among the three groups (Figure 2). Based on the KEGG annotation, lipid metabolism-related DEGs and pathways were further analyzed. A total of 46 DEGs were enriched in 14 lipid metabolism-related pathways (Table 4). Among them, LAvsMA contained 10 pathways and 16 DEGs; MAvsHA contained 14 pathways and 36 DEGs; LAvsHA contained 14 pathways and 46 DEGs. Significance analysis revealed that sphingolipid metabolism (ko00600), glycerol metabolism (ko00561), steroid hormone biosynthesis (ko00140), and primary bile acid biosynthesis (ko00120) were significantly enriched. There were 16 DEGs in the four significantly enriched pathways. Due to the crude lipid content was continuously rising, further trend analysis of the screened DEGs was necessary. Trend analysis revealed that *PLPP1a*, *PLPP2b*, and *CYP21a2* were continuously upregulated, *AKR1D1*, *UGT1a1*, and *CYP1a* were continuously downregulated. The relationships between six genes and the four significantly enriched pathways are shown in Table 5.

**TABLE 4:** The fourteen lipid metabolism-related pathways.

Pathway	*p*-value	Ko
Sphingolipid metabolism	0.002843	Ko00600
Glycerine metabolism	0.01974	Ko00561
Steroid hormone biosynthesis	0.041708	Ko00140
Primary bile acid biosynthesis	0.048403	Ko00120
Ether lipid metabolism	0.070951	Ko00565
Glycerophospholipid metabolism	0.131206	Ko00564
Fatty acid degradation	0.378736	Ko00071
Fatty acid biosynthesis	0.455371	Ko00061
Steroid biosynthesis	0.486033	Ko00100
Linoleic acid metabolism	0.500713	Ko00591
Alpha-linolenic acid metabolism	0.500713	Ko00592
Arachidonic acid metabolism	0.513544	Ko00590
Fatty acid elongation	0.676847	Ko00062
Biosynthesis of unsaturated fatty acids	0.703803	Ko01040

**TABLE 5:** Significantly enriched pathways and DEGs.

Pathway	DEGs	*p*-value	Ko
sphingolipid metabolism	*PLPP1a*↑ *PLPP2b*↑	0.002843	Ko00600
glycerine metabolism	*PLPP1a*↑ *PLPP2b*↑	0.01974	Ko00561
steroid hormone biosynthesis	*AKR1D1*↓ *UGT1a1*↓ *CYP1a*↓ *CYP21a2*↑	0.041708	Ko00140
primary bile acid biosynthesis	*AKR1D1*↓	0.048403	Ko00120

Note: ↑ represents upregulation and ↓ represents downregulation.

### 3.3 Metabolite extraction and LC-MS/MS analysis

**FIGURE 3:**
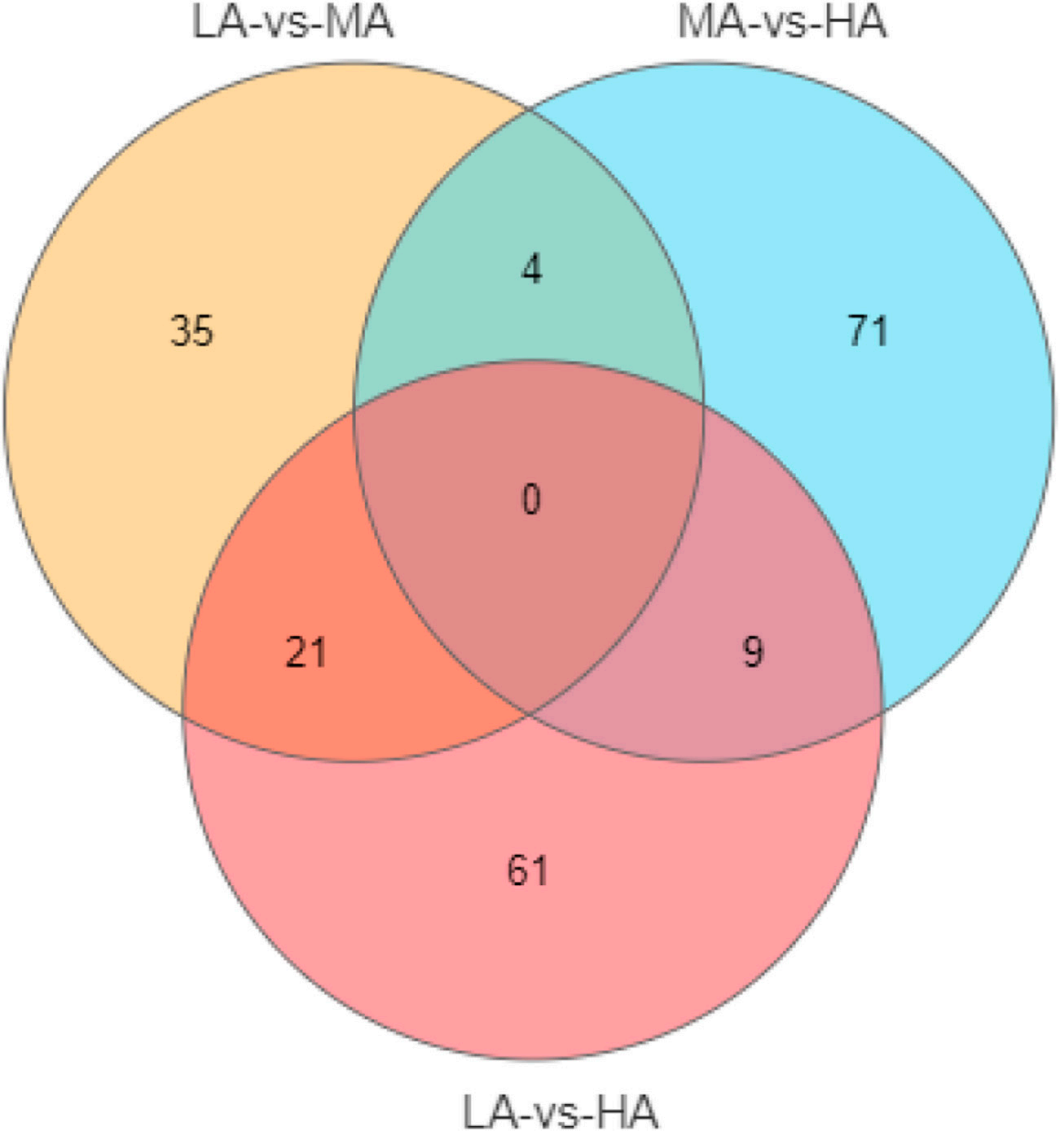
The venn of different metabolites distribution in three groups.

According to analyses of the metabolites, 10878 metabolites and 8540 metabolites were obtained in positive and negative modes, respectively. Based on the variable important in projection (VIP) values of OPLS-DA, the significantly different metabolites were screened based on a VIP ≥1 and *p* ≤ 0.05. In negative ion modes, a total of 201 different metabolites were detected in the three groups (Figure 3). To explore the lipid metabolism pathways, we selected different lipid-relevant metabolites for KEGG enrichment analysis. The five lipid metabolism pathways were mainly related to arachidonic acid metabolism, primary bile acid biosynthesis, unsaturated fatty acid biosynthesis, sphingolipid metabolism, and steroid hormone biosynthesis. There were six metabolites identified in the five pathways: eicosapentaenoic acid, sphinganine, 11-beta-hydroxyprogesterone, hydroxyeicosatetraenoic acid, cholic acid, and glycochenodeoxycholate (Table 6). In positive ion modes, no metabolites were detected.

**TABLE 6:** Significantly enriched pathways and differential metabolites.

Pathway	Metabolites	*p*-value	Ko
Primary bile acid biosynthesis	Cholic acid↓ glycochenodeoxycholate↑	0.011181	Ko00120
Sphingolipid metabolism	Sphinganine↑	0.103208	Ko00600
Steroid hormone biosynthesis	11-beta-hydroxyprogesterone↑	0.166955	Ko00140
Biosynthesis of unsaturated fatty acids	Eicosapentaenoic acid↑	0.212247	Ko01040
Arachidonic acid metabolism	Hydroxyeicosatetraenoic acid↑	1	Ko00590

Note: ↑ represents upregulation and ↓represents downregulation.

### 3.4 Integrative transcriptome and metabolome analysis

To screen for DEGs and different metabolites associated with lipid metabolism, integrated transcriptome and metabolome analyses were performed using the O2PLS, correlation coefficient models and KEGG pathway. The common KEGG pathways were analyzed for the differential genes and metabolites. Integrative analysis revealed that primary bile acid and steroid hormone biosynthesis were enriched in both the transcriptome and metabolome.

**FIGURE 4:**
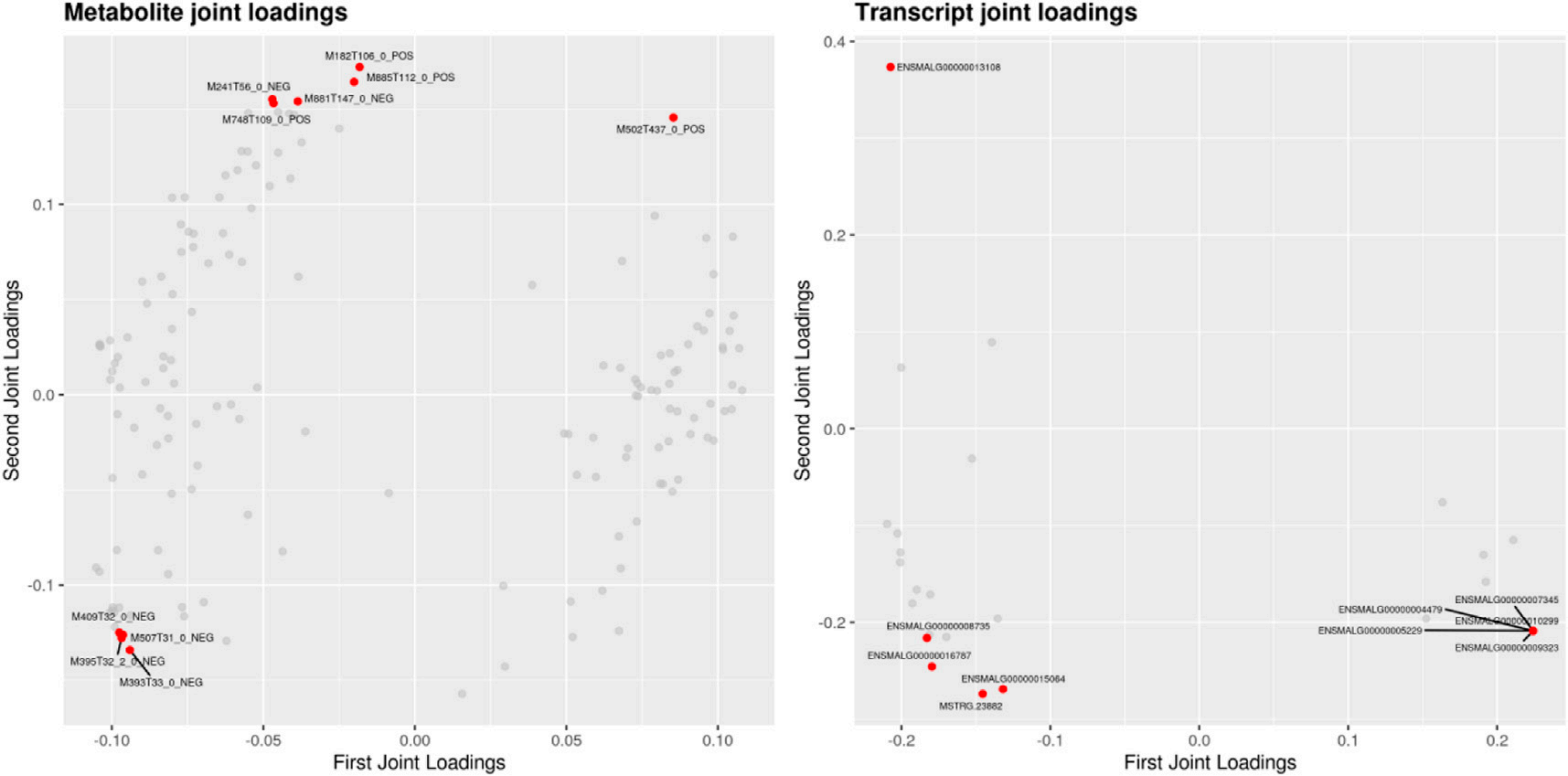
Loading plots for the differential metabolites and differential genes. Note: loading value represents the explanatory ability of a variable (metabolite/gene) in each component (that is, the contribution to the difference between groups). The positive and negative loading value represents the positive or negative correlation with another group. The greater the absolute value of the load, the stronger the correlation.

**FIGURE 5:**
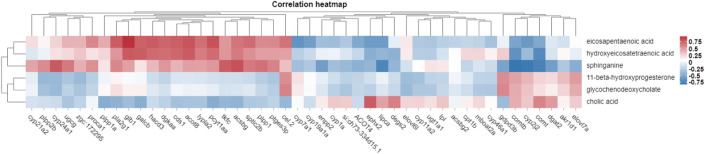
Heat plot showing the correlations between differential metabolites and differential genes. Note: red and blue indicate positive and negative correlations between the transcriptomics and metabolomics data, respectively.

**FIGURE 6:**
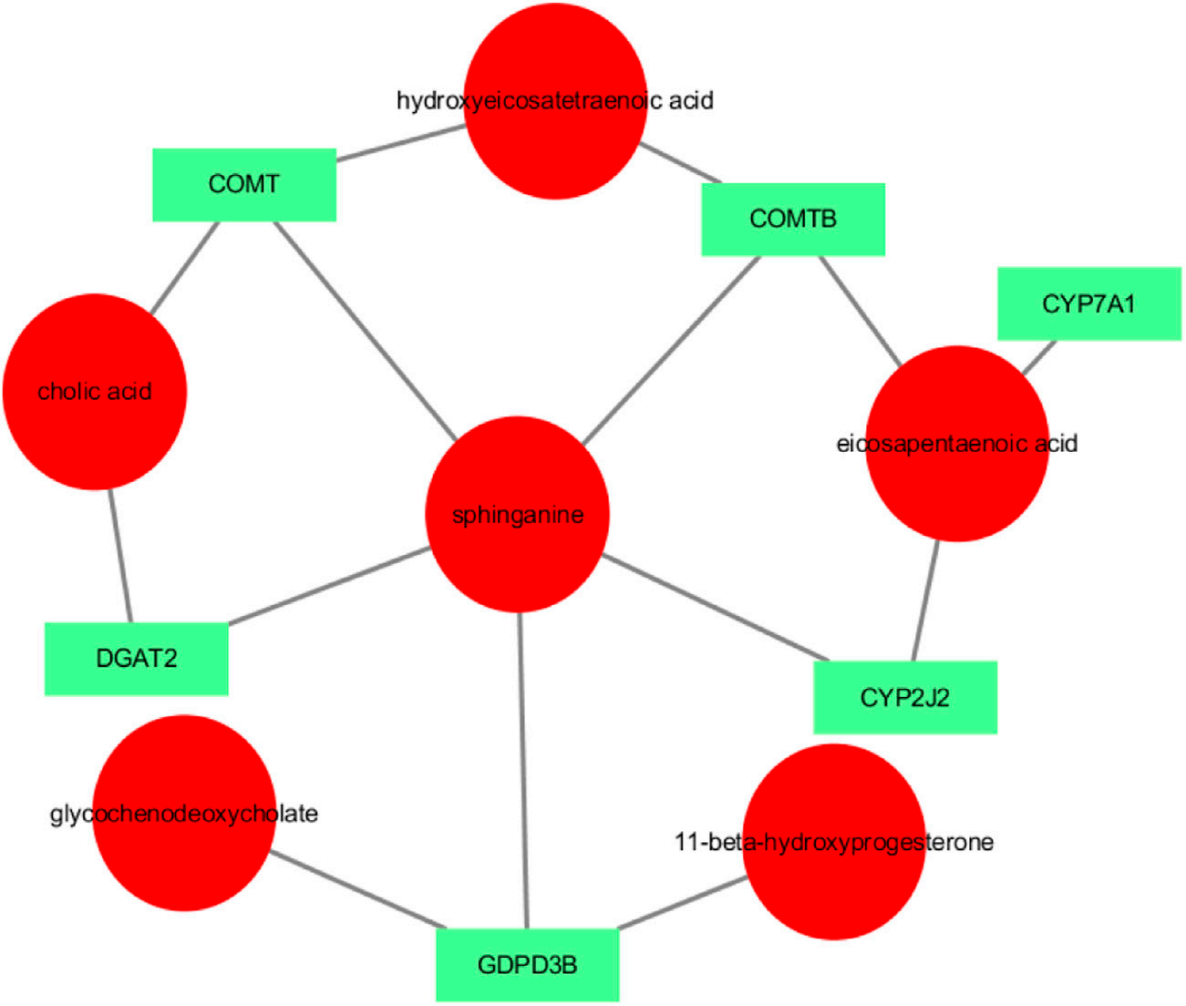
Network showing the correlations between metabolites and genes. Note: circles represent metabolites and squares represent genes. The absolute value of pearson coefficient ≥0.5.

Transcriptomic and metabolomic data were correlated analysis by O2PLS model. Components of the O2PLS model were calculated and reflected the contribution of the transcriptome and metabolome to the total variation. Therefore, the O2PLS model was very reliable for explaining the total variation in the comprehensive analysis of transcriptome and metabolome.The combined loading diagram of metabolites and transcripts was constructed according to the loading values shown in Figure 4. High absolute loading values indicated a strong correlation between the differential genes and differential metabolites. DEGs and differential metabolites farther from the origin were more closely related to each other. The correlation between metabolites and genes were evaluated by calculating pearson coefficients. The correlation matrix of the heat map revealed negative (blue) and positive (red) correlations between lipid-related differential metabolites and genes (Figure 5). Correlation network maps showed lipid-related differential genes or metabolites at important associated positions (Figure 6). Sphinganine was the main associated metabolite and it was correlated with five DEGs.

## 4 Discussion

Currently, the effects of VA on lipid metabolism in fish have been reported, however there are few studies on its regulatory mechanism. Therefore, this study aims to explore the possible mechanism of VA-mediated regulation of lipid metabolism through comprehensive analysis of transcriptomics and metabolomics.

### 4.1 VA and bile acid biosynthesis

The primary bile acid biosynthesis pathway was significantly enriched in both the transcriptome and metabolome, indicating that there is a complex interaction between VA and bile acids. Retinoic acid receptor (*RAR*), farnesoid X receptor (*FXR*), and retinoid X receptor (*RXR*) are the main regulators of VA and bile acid homeostasis (Zoubek et al., 2017). In the liver, VA and its metabolites activate *RAR* and *RXR*, generate *RAR/RXR* heterodimers, and regulate bile acid synthesis and metabolism (Li et al., 2021). In addition, retinoic acid can inhibit the genes involved 5in bile acid synthesis (Mamoon et al., 2014), in which RAR and *RXR* may be involved. Studies have shown that VA inhibits the expression of the bile salt synthesis rate-limiting enzyme *CYP7A1*, thereby reducing bile generation (Jahn et al., 2016). In this study, *AKR1D1* was inhibited instead of *CYP7A1*. *AKR1D1* plays an important role in steroid metabolism (Gathercole et al., 2021), but biologically, it is better known for its involvement in bile acid biosynthesis, where it catalyzes a key step that introduces the 5β-configuration into primary bile acids (Russell, 2009). This decrease in cholic acid levels may be due to the inhibition of *AKR1D1*. Another reason is that cholic acid is used to produce 12α-hydroxylated bile acid, which lead to hepatic lipid accumulation (Maegawa et al., 2021). In contrast, the glycochenodeoxycholate content was found to be increased in the metabolome. Glycochenodeoxycholate is a hydrophobic bile salt and that can cause lipid accumulation in the liver (Chen and Chen, 2014). Simultaneously, glycochenodeoxycholate can increase lipid solubility and promote intestinal absorption (Wu et al., 2016). Notably, high concentrations of glycochenodeoxycholate induced hepatocyte apoptosis (Wang et al., 2005). This may also explain why high levels of liver fat are disadvantageous in fish.

### 4.2 VA and fatty acid synthesis

Fatty acids are components of various molecules, including phospholipids, sphingolipids, and esters. The regulation of fatty acid metabolism by VA has been confirmed in other studies (Chen and Chen, 2014; Blaner, 2019). The genes for fatty acid extension, desaturation and synthesis pathways can be regulated by VA. This is achieved through retinoic acid (RA), a product of VA metabolism.

The first step in fatty acid synthesis is the conversion of acetyl-CoA to malonyl-CoA by acetyl-CoA carboxylase. It is then catalyzed by fatty acid synthase to form palmitic acid. Palmitic acid can be prolonged and desaturated to produce fatty acids with longer chains and double bonds (Kihara, 2012; Zhang et al., 2016). Elongases add two carbon atoms each time to create longer-chain fatty acids. Desaturases introduce double bonds into saturated and unsaturated fatty acids, resulting in the production of monounsaturated and polyunsaturated fatty acids, respectively. The mechanism of VA regulate fatty acid synthesis has been extensively studied. Studies have shown that when VA deficiency, the mRNA levels of acetyl-CoA carboxylase, fatty acid synthase and stearoyl-CoA desaturase 1 were reduced; when RA treatment, the mRNA levels were increased (Yang et al., 2021).

Ayuso et al. (2015) found that VA increases the content of polyunsaturated fatty acids in pork. Similar to the results of our study, the levels of the polyunsaturated fatty acids, eicosapentaenoic and hydroxyeicosatetraenoic acid, increased significantly. This is probably because of the increase in stearoyl-CoA desaturase 1. Stearoyl-CoA desaturase 1 was responsible for the formation of double bond and regulation of lipogenesis in the process of fatty acid synthesis (Koeberle et al., 2016; ALJohani et al., 2017). RA could induce stearoyl-CoA desaturase 1 mRNA expression (Miller et al., 1997) and desaturase index in adipose tissues (Gorocica-Buenfil et al., 2008).

### 4.3 VA and phospholipid synthesis

Oliveros et al. (2007) found that VA deficiency induced a hypolipidemic effect by decreasing the total phospholipid content in the liver, owing to low phosphatidylcholine synthesis and enhanced fatty acid oxidation in rats, and this was consistent with the results of our study. In the present study, PLPP1a and PLPP2b were significantly upregulated with increasing VA content. PLPP1a and PLPP2b are phospholipid phosphatases that belong to the lipid phosphate phosphatase family. Lipid phosphate phosphatase has wide substrate selectivity and can hydrolyze a variety of lipid phosphoric acids containing single lipid bonds, including phosphatidic acid, lysophosphatidic acid, diacylglycerol pyrophosphate, and sphingosine 1-phosphate, and it participates in the synthesis of glycerol (Toke et al., 1998; Li et al., 2017; Tang and Brindley, 2020). The significant increase in sphinganine levels in the metabolome may be related to the hydrolysis of sphingosine 1-phosphate.

### 4.4 VA and steroid hormone synthesis

VA is essential for the synthesis and secretion of steroid hormones. The expression of 11β-hydroxysteroid dehydrogenase type 1 was increased by VA deficiency in the hypothalamus (C62.5%) and hippocampus (C104.7%) (Marissal-Arvy et al., 2013). RA treatment could reverse the effects of VA deficiency on 11β-hydroxysteroid expression. The inhibitory effect of retinoids on 11β-hydroxysteroid dehydrogenase type 1 expression has been demonstrated peripherally in the liver and visceral fat of WNIN/Ob obese rats (Sakamuri et al., 2011), in the adipose tissue of mice (Sakuta et al., 2006), and in myotubes *in vitro* (Aubry and Odermatt, 2009). In contrast, in this study, the level of 11β-hydroxyprogesterone was increased by VA, but this discrepancy may be species related. Within the cell, 11β-hydroxysteroid dehydrogenase type 1 catalyzes the regeneration of active glucocorticoids, thereby amplifying their action (Marissal-Arvy et al., 2013). Similarly, in our study, we found that *CYP21a2* was associated with glucocorticoid synthesis (Heidelberg, 2008) which was significantly upregulated.

In this study, comparative metabolomics and transcriptomics were applied to investigate lipid metabolite changes in rice field eel when fed different levels of VA. Comparative transcriptome analysis revealed that VA affected multiple lipid metabolic pathways, especially sphingolipid and glycerin metabolism, and steroid hormone and primary bile acid biosynthesis. Metabolite analysis revealed that the levels of lipids, such as eicosapentaenoic acid, sphinganine, 11-beta-hydroxyprogesterone, hydroxyeicosatetraenoic acid, and glycochenodeoxycholate, increased significantly. Integrative analyses of the transcriptome and metabolome revealed a significant correlation between differential metabolites and differential genes, suggesting that these are the main metabolic pathways. These findings provide reliable models at the transcriptional and metabolic levels which will facilitate further investigation into the effects of VA on lipid metabolism.

## 5 Conclusion

In conclusion, VA is found to promote lipid deposition in rice field eel. The most important pathway is primary bile acid synthesis and *AKR1D1* plays a key role in this process. Integrated transcriptome and metabolome analyses have revealed that sphingosine is the main form of lipid deposits. Future work can be focused on the primary bile acid synthesis pathway, *AKR1D1* gene and sphingosine.

## Data Availability

The datasets presented in this study can be found in online repositories. The names of the repository/repositories and accession number(s) can be found in the article/Supplementary material.
